# Pathological characteristics and prognosis of very young patients with early breast cancer: a comparative analysis with older patients

**DOI:** 10.2340/1651-226X.2026.45584

**Published:** 2026-06-29

**Authors:** Youri Park, Jin Hoon Nam, Yeonjoo Kwon, Jung Ho Park, Sanghwa Kim, Young Ah Lim, Hee-Joon Kang, Doyil Kim, Janghee Lee

**Affiliations:** a Department of Surgery, Dongtan Sacred Heart Hospital, Hallym University, Hwaseong-Si, Republic of Korea; b Department of Surgery, Ewha Womans University Mokdong Hospital, Ewha Womans University College of Medicine, Seoul, Republic of Korea; c Department of Surgery, Hallym University Sacred Heart Hospital, Hallym University, Hwaseong-Si, Republic of Korea

**Keywords:** breast cancer, age, pathologic characteristics, prognosis, locoregional recurrence

## Abstract

**Background and purpose:**

It has generally been established that younger breast cancer patients exhibit unfavorable biology and poorer prognosis. However, previous studies have not consistently demonstrated differences between younger and older patients. The objective of this study, therefore, was to compare pathological characteristics and prognosis in young patients (age ≤ 40 years) with early breast cancer with those of older patients (age ≥ 55 years).

**Patients/material and methods:**

Individuals diagnosed with early breast cancer (age ≤ 40 years) and those ≥ 55 years of age, who were treated at two hospitals between June 2003 and December 2019, were identified. Oncological outcomes, including recurrence-free survival (RFS), distant RFS (DRFS), and locoregional RFS (LRRFS), were compared between two groups. Kaplan–Meier survival curves and the Cox proportional hazards model were used to evaluate.

**Results:**

Data from 288 young (≤ 40 years) and 830 older (≥ 55 years) patients were included. Pathologically, younger patients exhibited a higher prevalence of high-grade tumors. There was no significant difference in RFS and DRFS between the younger and older patient groups (RFS: hazard ratio 1.11 [95% confidence intervals (CI) 0.74–1.66]; *P* = 0.613; DRFS: hazard ratio 1.03 [95% CI 0.65–1.62]; *P* = 0.897). However, LRRFS was unfavorable in younger patients (hazard ratio, 2.15; 95% CI, 1.03–4.49; *P* = 0.043). Subgroup analysis revealed a significant difference in LRRFS among hormone receptor (HR)-positive/human epidermal growth factor receptor 2 (HER2)-negative breast cancer patients (hazard ratio 4.04 [95% CI 1.17–13.97]; *P* = 0.027); however, no significant difference was observed in other subtypes.

**Interpretation:**

Younger patients with breast cancer exhibited a higher frequency of high-grade tumors, although no differences were observed in RFS and DRFS. However, locoregional recurrence was more common among younger patients, especially HR-positive/HER2-negative breast cancer.

## Introduction

Breast cancer is the most common malignant tumor among women, with 2.3 million new cases diagnosed worldwide in 2022 [1]. Asian countries have experienced a more rapid increase in breast cancer incidence over the past few decades than that in Western countries [2]. Furthermore, in Asian countries, the peak age of breast cancer incidence is the 40s, unlike in Western countries, where the peak age is in the 60s and 70s [3]. This highlights the distinct characteristics of a higher proportion of premenopausal young patients with breast cancer in the Asian population.

Age is a known factor associated with the genetic and pathological characteristics and prognosis of breast cancer. Previous studies have reported that young patients with breast cancer tend to exhibit unfavorable pathological features, such as high-grade tumors and lymphovascular invasion (LVI), as well as aggressive subtypes such as human epidermal growth factor receptor 2 (HER2)-positive or triple negative breast cancer (TNBC) [4–6]. They are also more likely to harbor genetic factors associated with hereditary breast cancer such as *BRCA1/2* mutations [7, 8]. Consequently, young(er) age is considered to be an independent risk factor for breast cancer recurrence and mortality [9–12].

Nevertheless, several studies have argued that young patients with breast cancer do not always experience inferior oncological outcomes compared with their older counterparts [13]. Some authors have suggested that the impact of age on prognosis may vary depending on the type of breast cancer [14–17]. Moreover, current breast cancer guidelines, with minor variations, generally recommend the same treatment regardless of age [18].

The purpose of the present study was to compare the clinicopathological features of young(er) patients with breast cancer (≤ 40 years of age), who are mostly premenopausal, with those of older postmenopausal patients (≥ 55 years of age) and to analyze their oncological outcomes. Additionally, we sought to identify the subgroups in which young(er) age may be a high-risk factor.

## Methods

### Study population

Data from patients diagnosed with and treated for primary breast cancer at Dongtan Sacred Heart Hospital and Hallym Sacred Heart Hospital (Gyeonggi, South Korea) between June 2003 and December 2019 were retrospectively collected and analyzed. To clearly compare the prognosis and characteristics of very young patients with those of older, postmenopausal patients, we divided the cohort into two groups based on age at diagnosis: younger (≤ 40 years) and older (≥ 55 years). Patients aged > 40 and < 55 years were excluded, as these groups were considered to include a high proportion of pre- or perimenopausal individuals. The clinicopathological features, treatments, and recurrence information of each patient were manually collected through a review of medical charts. Patients with de novo stage IV disease were excluded from the study.

### Clinicopathological features

Clinicopathological patient information was obtained through a comprehensive review of medical records. For tumor size and lymph node (LN) metastasis, the results of the final surgical specimens were used in patients treated in the adjuvant setting. For patients who underwent neoadjuvant systemic therapy, these factors were assessed based on the clinical stage determined by pretreatment imaging studies. For LN metastasis, patients were classified as positive if metastasis was confirmed by biopsy or was suspected on the basis of imaging studies, even if it is not confirmed by biopsy. Menopausal status was determined based on patient interviews and follicle-stimulating hormone test results, while pathological factors were assessed using the results from the local laboratories of each participating hospital. The estrogen receptor (ER), progesterone receptor (PR), and HER2 were evaluated using immunohistochemistry and in situ hybridization, particularly for HER2. Their expression is reported as either positive or negative.

### Statistical analysis

The endpoints for oncological outcomes included recurrence-free survival (RFS), distant RFS (DRFS), and locoregional RFS (LRRFS). RFS refers to the period after treatment when a patient remains free of tumor recurrence; DRFS was defined as the interval from treatment to first distant recurrence (DR), whereas LRRFS was defined as the interval from treatment until first locoregional recurrence (LRR). DR was characterized by metastasis to other organs, excluding contralateral breast cancer. Local recurrence was defined as recurrence in the ipsilateral breast, chest wall, or skin, while regional recurrence was defined as recurrence in the ipsilateral LN. To compare the clinicopathological features of patients ≤ 40 and those ≥ 55 years of age, the chi-squared test was used for categorical variables, and the independent two-sample *t*-test was used for continuous variables. Kaplan–Meier survival curves with log-rank tests were used to evaluate oncological outcomes in young(er) and older patients. Univariate and multivariate analyses of prognostic risk factors were performed using the Cox proportional hazards model for the entire cohort and subgroups. We performed multivariate analyses for RFS and DRFS, including seven clinicopathologic factors along with age and chemotherapy as variables. For LRRFS, to achieve a more refined analysis, we first conducted univariate analyses for each of the eight variables and then selected the four variables with *P* < 0.050 for stepwise multivariate analysis. All statistical tests were two-sided, and *P* < 0.050 was considered to be statistically significant. Statistical analyses were performed using SPSS version 27.0 (IBM Inc., Armonk, NY, USA) and Prism version 9 (GraphPad Inc., San Diego, CA, USA).

## Results

### Baseline characteristics

**Figure 1:**
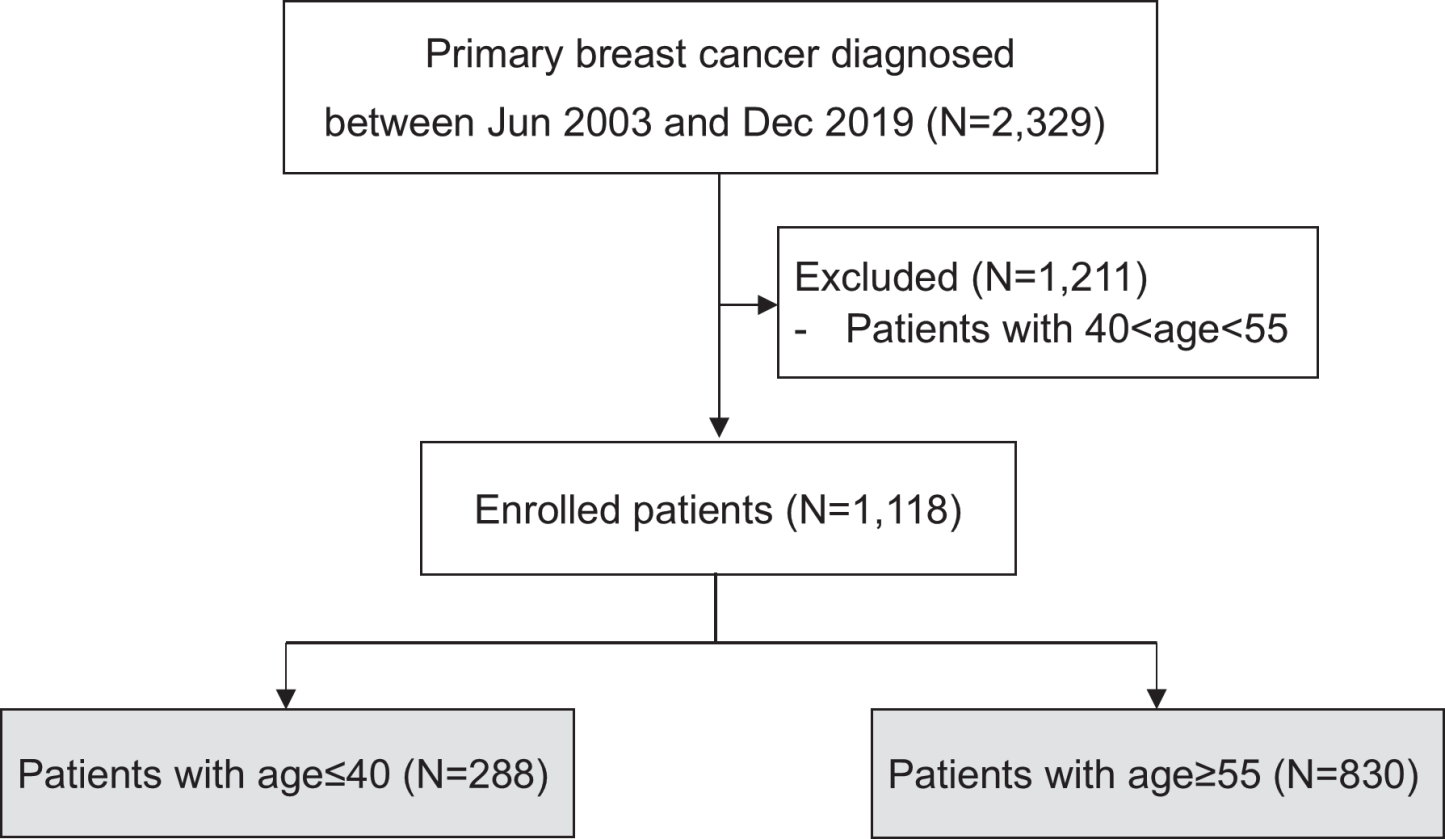
Consort diagram of enrolled patients.

Initially, data from 2329 potentially eligible patients diagnosed with primary breast cancer during the study period were retrospectively collected (Figure 1). Among these, 1211 patients aged 40–55 years were excluded, leaving 1118 patients, who were divided into two groups for comparative analysis based on age: young(er) (≤ 40 years [*n* = 288]) and older (≥ 55 years [*n* = 830]).

Clinicopathological characteristics of the two groups are summarized in Table 1. The mean age of the younger and older patients was 36.0 years (98.6% premenopausal) and 64.0 years (98.3% postmenopausal), respectively. There were no statistically significant differences between the two groups in terms of breast surgery method, stage including tumor size, LN metastasis, breast cancer subtype, and LVI. However, younger patients had a higher proportion of high-grade cancers (*P* = 0.001) and underwent neoadjuvant and adjuvant chemotherapy more frequently than older patients (*P* < 0.001). In contrast, there were no differences between the two groups in the use of endocrine therapy and anti-HER2 therapy.

**Table 1:** Baseline characteristics of enrolled patients.

Variables	Young patients (≤ 40) (*N* = 288)	Old patients (≥ 55) (*N* = 830)	*P*
Breast surgery			0.684
BCS	204 (70.8)	593 (71.4)	
Mastectomy	84 (29.2)	235 (28.3)	
None	0 (0.0)	2 (0.2)	
Tumor size (mm)			0.257
≤ 20	156 (54.2)	483 (58.2)	
> 20	131 (45.5)	347 (41.8)	
Unknown	1 (0.3)	0 (0.0)	
LN metastasis			0.269
Negative	177 (61.7)	542 (65.3)	
Positive	110 (38.3)	288 (34.7)	
Stage			0.228
I	115 (39.9)	355 (42.8)	
II	134 (46.5)	385 (46.4)	
III	38 (13.2)	90 (10.8)	
Unknown	1 (0.3)	0 (0.0)	
Subtype			0.497
HR-positive/HER2-negative	149 (51.7)	455 (54.8)	
HER2-positive	74 (25.7)	209 (25.2)	
TNBC	64 (22.2)	159 (19.2)	
Unknown	1 (0.3)	7 (0.8)	
HG			0.001
I or II	161 (55.9)	553 (66.6)	
III	107 (37.2)	228 (27.5)	
Unknown	20 (6.9)	49 (5.9)	
LVI			0.315
Negative	188 (65.3)	584 (70.4)	
Positive	83 (28.8)	221 (26.6)	
Unknown	17 (5.9)	25 (3.0)	
Chemotherapy			< 0.001
Not performed	23 (8.0)	162 (19.5)	
Neoadjuvant	30 (10.4)	48 (5.8)	
Adjuvant	234 (81.3)	618 (74.5)	
Unknown	1 (0.3)	2 (0.2)	
Endocrine therapy			0.158
Not performed	80 (27.8)	209 (25.2)	
Performed	207 (71.9)	621 (74.8)	
Unknown	1 (0.3)	0 (0.0)	
Anti-HER2 therapy			0.236
Not performed	227 (78.8)	655 (78.9)	
Performed	60 (20.8)	175 (21.1)	
Unknown	1 (0.3)	0 (0.0)	

BCS: breast-conserving surgery; LN: lymph node; HR: hormone receptor; HER2: human epidermal growth factor receptor 2; TNBC: triple negative breast cancer; HG: histologic grade; LVI: lympho-vascular invasion.

### Oncological outcomes according to age group

**Figure 2:**
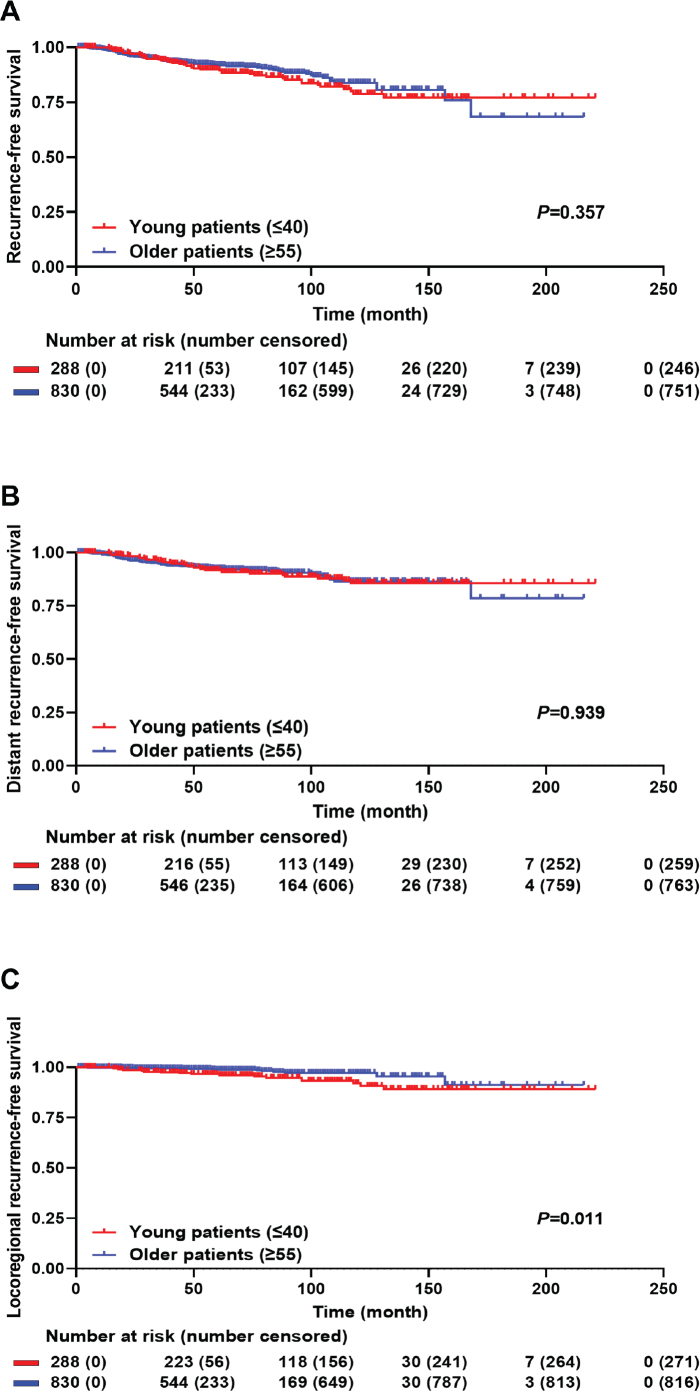
Kaplan-Meier survival curve for oncologic outcomes in young patients and older patients. (A) RFS (*P* = 0.357). (B) DRFS (*P* = 0.939). (C) LRRFS (*P* = 0.011). RFS: recurrence-free survival; DRFS: distant recurrence-free survival; LRRFS: locoregional recurrence-free survival.

The median follow-up for the younger patients was 88 months (range, 0–220 months), with a 5-year RFS rate of 91.0%. During the follow-up period, a total of 46 recurrence events occurred in 42 patients, including 17 LRR events and 29 DR events. Median time to relapse was 47 months (Q1–Q3, 28–80 months). For older patients, the median follow-up period was 63 months (range, 0–216 months), and the 5-year RFS rate was 92.9%. During the follow-up period, a total of 81 recurrence events occurred in 79 patients, including 14 LRR events and 67 DR events. Median time to relapse was 35 months (Q1–Q3, 18–62 months). Kaplan–Meier survival analysis revealed no significant difference in RFS between younger and older patients (*P* = 0.357) (Figure 2A). Similarly, there was no statistical difference in DRFS between the younger and older age groups (*P* = 0.939) (Figure 2B). In contrast, LRRFS was worse in younger patients than that in older patients (*P* = 0.011) (Figure 2C).

In multivariate analysis, younger age was not identified as a poor prognostic factor for RFS (hazard ratio 1.11 [95% confidence interval (CI) 0.74–1.66]; *P* = 0.613) and DRFS (hazard ratio 1.03 [95% CI 0.65–1.62]; *P* = 0.897) (eTable 1). Tumor size, LN metastasis, and LVI were significant factors for RFS, whereas tumor size and LN metastasis were associated with DRFS. However, younger age remained a risk factor for LRR (hazard ratio 2.15 [95% CI 1.03–4.49]; *P* = 0.043) (Table 2). Additionally, the TNBC subtype was also identified as another significant factor for LRRFS (hazard ratio 3.39 [95% CI 1.42–8.09]; *P* = 0.006).

**Table 2:** Univariate and multivariate analyses of risk factors for LRRFS.

Variables	Univariate		Multivariate	
	Hazard ratio (95% CI)	*P*	Hazard ratio (95% CI)	*P*
Age				
≥ 55	Ref.*		Ref.	
≤ 40	2.47 (1.21-5.07)	0.014	2.15 (1.03-4.49)	0.043
Breast surgery				
BCS	Ref.			
Mastectomy	0.94 (0.44-2.02)	0.881		
Tumor size (mm)				
≤ 20	Ref.		Ref.	
> 20	2.12 (1.04-4.33)	0.039	1.86 (0.88-3.90)	0.103
LN metastasis				
Negative	Ref.			
Positive	1.89 (0.93-3.82)	0.078		
Subtype				
HR-positive/HER2-negative	Ref.		Ref.	
HER2-positive	1.50 (0.60-3.73)	0.386	1.70 (0.66-4.42)	0.274
TNBC	2.85 (1.25-6.48)	0.012	3.39 (1.42-8.09)	0.006
HG				
I or II	Ref.		Ref.	
III	2.84 (1.36-5.95)	0.006	1.78 (0.78-4.03)	0.171
LVI				
Negative	Ref.			
Positive	1.82 (0.88-3.75)	0.106		
Chemotherapy				
Not performed	Ref.			
Performed	1.28 (0.39-4.24)	0.683		

* Reference value.

LRRFS: locoregional recurrence-free survival; BCS: breast-conserving surgery; LN: lymph node; HR: hormone receptor; HER2: human epidermal growth factor receptor 2; TNBC: triple negative breast cancer; HG: histologic grade; LVI: lympho-vascular invasion.

### Impact of young(er) age in subgroup analysis

**Figure 3:**
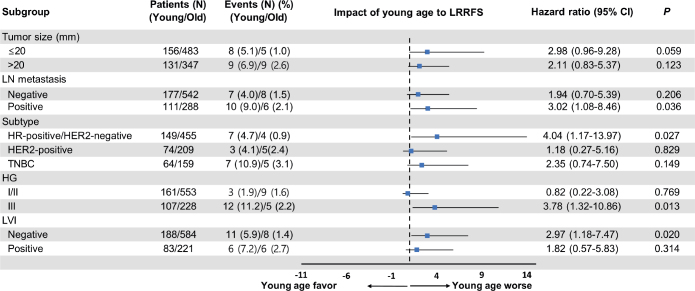
Forest plot of subgroup analysis on the effect of young age on LRRFS. LRRFS: locoregional recurrence-free survival; CI: confidence intervals; LN: lymph node; HR: hormone receptor; HER2: human epidermal growth factor receptor 2; TNBC: triple negative breast cancer; HG: histologic grade; LVI: lympho-vascular invasion.

Oncological outcomes based on age were further analyzed by dividing the patients into subgroups according to pathological features. Subgroup analyses based on tumor size, LN metastasis, breast cancer subtype, histological grade (HG), and LVI revealed that young age was not a risk factor for RFS or DRFS in most subgroups (eTable 2). In the LVI-negative group, young patients uniquely exhibited poorer RFS (hazard ratio 1.67 [95% CI 1.02–2.74]; *P* = 0.042). In contrast, younger age tended to be associated with poorer LRRFS across most subgroups (Figure 3). Specifically, in cases with LN metastasis, hormone receptor (HR)-positive/HER2-negative subtype, high HG, and negative LVI, young age was statistically linked to higher rates of LRR (LN-positive, hazard ratio 3.02 [95% CI 1.08–8.46]; *P* = 0.036; HR-positive/HER2-negative, hazard ratio 4.04 [95% CI 1.17–13.97]; *P* = 0.027; HG III, hazard ratio 3.78 [95% CI 1.32–10.86]; *P* = 0.013; LVI-negative, hazard ratio 2.97 [95% CI 1.18–7.47]; *P* = 0.020).

## Discussion

Our study found that young(er) patients with breast cancer exhibited no significant differences in most clinicopathological features, such as tumor size, LN metastasis, subtype, and surgical method, compared with older patients. However, high-grade tumors were significantly more common among younger patients. Furthermore, age was not a significant risk factor for overall or DR. Younger patients exhibited a higher incidence of LRR than their older counterparts, with the difference being particularly pronounced in patients with HR-positive/HER2-negative subtypes or high-grade tumors. However, some studies, particularly those conducted in Asian populations, have adopted 35 years as the cut-off. Park YH et al., in a study of Korean patients, defined young breast cancer as age ≤ 35 years and, unlike our findings, reported poor RFS and overall survival compared with patients older than 35 years. In addition, Shin DS et al. reported that, among patients aged ≤ 45 years had a higher incidence of late DR beyond 5 years. These findings suggest that the choice of age cur-off for defining young breast cancer patients can influence study outcomes and should be carefully considered when interpreting related research.

Asian countries are known to have a younger peak age of breast cancer incidence than Western countries [3]. In China, the median age of patients diagnosed with breast cancer between 2011 and 2021 was 47.0 years, with approximately 23.8% < 40 years of age [19]. Similarly, an analysis of the Taiwan Cancer Database from 2002 to 2006 revealed that the median age of patients with breast cancer was 49.4 years, with approximately 14.2% of patients < 40 years of age [20]. In the Republic of Korea, approximately 12.0% of patients with breast cancer between 2010 and 2015 were < 40 years of age [21]. In our cohort, patients < 40 years of age accounted for approximately 10.1% of all breast cancer cases, which is significantly higher than the 4–5% typically reported in Western countries, including the United States [22, 23].

There is currently no universally accepted age cut-off for defining young breast cancer patients, and the criteria have varied across studies. Many studies, including ours, have used 40 years as the threshold [9, 12, 14–16]. However, some studies, particularly those conducted in Asian populations, have adopted 35 years as the cut-off. Park YH et al., in a study of Korean patients, defined young breast cancer as age ≤ 35 years and, unlike our findings, reported poor RFS and overall survival compared with patients older than 35 years [24]. In addition, Shin DS et al. reported that, among patients aged ≤ 45 years had a higher incidence of late DR beyond 5 years [25]. These findings suggest that the choice of age cut-off for defining young breast cancer patients can influence study outcomes and should be carefully considered when interpreting related research.

Young patients with breast cancer exhibit more aggressive pathological features than those by older patients. A United Kingdom-based observational study involving 2956 patients ≤ 40 years of age reported that more than one-half of these patients had high-grade tumor(s), and one-third were ER-negative [26]. Additionally, in an analysis of 200,000 patients with breast cancer using the SEER database, Gnerlich et al. reported that patients < 40 years of age not only exhibited more aggressive tumor biology but also had larger tumor sizes and higher rates of LN metastasis [9]. Furthermore, multiple studies using molecular subtyping with gene expression profiling, such as PAM50, have shown that younger patients are more likely to have basal-like or HER2-enriched tumors, whereas luminal-A tumors are less common [12, 27]. In our cohort, younger patients exhibited a higher prevalence of high-grade tumors, which is consistent with previous studies, despite the lack of differences in stage or subtype between younger and older patients.

Nevertheless, there are conflicting opinions regarding the differences in prognoses between younger and older patients, particularly in cases of HER2-positive or TNBC. Previous studies reported higher recurrence rates in younger patients [9, 11]. However, paradoxically, many reports indicate that this difference is unclear in aggressive subtypes such as HER2-positive and TNBC. Azim et al. reported that patients ≤ 40 years of age had worse RFS compared with those > 40 years, but no statistically significant difference was observed in HER2-positive and TNBC subtypes in an analysis of 39 published datasets of early breast cancer [12]. Furthermore, in a retrospective analysis of the HERceptin Adjuvant study, a large randomized controlled trial focused on HER2-positive breast cancer and reported that age was not a strong risk factor for early recurrence [16]. A retrospective study involving approximately 2000 Asian participants also suggested that young age was not an independent risk factor for poor prognosis in HER2-positive and TNBC cases [14]. Consistent with these findings, our study failed to demonstrate an inferior prognosis in young patients with aggressive subtypes.

In contrast, in HR-positive/HER2-negative breast cancer, which has a favorable phenotype, young(er) age is considered a more distinct prognostic factor. Most studies analyzing the differences in recurrence between young and older patients have reported the most pronounced disparities in luminal breast cancer [11, 12, 14]. In particular, in LRR, young age is recognized as an independent risk factor, not only in patients who undergo breast-conserving surgery but also in those who undergo mastectomy [28–30]. Furthermore, young(er) patients have a higher proportion of high-risk scores on multigene assays, including Oncotype DX(Exact Sciences, Madison, WI, USA) or MammaPrint(Agendia NV, Amsterdam, The Netherlands) [31, 32], and studies report a higher incidence of late recurrence beyond 5 years [25], emphasizing the significance of young age as a critical prognostic factor in HR-positive/HER2-negative breast cancer. The results from our cohort also confirmed that younger patients had poorer LRRFS, which can be regarded as an extension of previous findings. However, our study did not demonstrate age-related difference in RFS and DRFS beyond LRRFS. Although the exact reason for this finding remains unclear, one possible explanation is that younger patients may have received more aggressive systemic treatment, such as chemotherapy. In addition, the median follow-up duration differed between the younger and older groups (88 months vs 63 months), and the overall follow-up period may have been relatively short to full capture the pattern of late recurrence, which is a characteristic feature of HR-positive/HER2-negative breast cancer.

In breast cancer, young age is strongly associated with premenopausal status. For this reason, we excluded patients between 40 and 55 years of age, who may have included pre- or perimenopausal individuals, and compared them with older patients aged ≥ 55 years to analyze the prognosis of younger patients with breast cancer. As a result, 98.6% of young patients in our cohort were premenopausal, whereas 98.3% of older patients were postmenopausal. Considering that the 40s and 50s are the peak age ranges for breast cancer incidence in Asia, many patients were excluded based on the age criterion. However, our study effectively controlled for the menopausal status, which could influence prognosis. This is a notable strength of our study, enabling a clear comparison of outcomes between young premenopausal and older postmenopausal patients.

One limitation of our study was the potential for selection bias due to its retrospective design. To mitigate this, we established strict age-based inclusion criteria and performed multivariate analysis. Additionally, differences in censoring patterns were observed between age groups, with higher early censoring in the younger cohort, which may reflect differences in clinical follow-up or loss to follow-up. While our median follow-up of 63–88 months provides robust data for early recurrences, it may be considered relatively short, given the generally favorable prognosis of breast cancer, potentially missing late recurrences particularly in HR-positive/HER2-negative subtypes, as mentioned earlier. We were also unable to assess genetic predispositions such as *BRCA 1/2* mutations. The proportion of hereditary breast cancers, including BRCA 1/2, is higher among young patients with breast cancer [6, 7]. Although there is currently insufficient evidence, suggesting that hereditary breast cancer is associated with poor outcomes, incorporating information on such genetic backgrounds could have enhanced our analysis. Future studies should address these issues to provide further insights. Furthermore, tumor proliferation markers such as the Ki-67 labeling index (LI) were not included in our analysis. This was because our cohort contained a substantial number of patients treated in earlier periods, leading to considerable missing data for Ki-67 LI, along with inter-institutional variability between the two centers. Therefore, we determined that a reliable analysis was not feasible. Future studies that incorporate centrally reviewed data on proliferation biomarkers may provide more robust and informative results. Finally, we were unable to include patient comorbidities or performance status as variables in the analysis of oncologic outcomes. Older patients are more likely to have comorbid conditions compared to younger patients, which may have introduced bias into the prognostic analysis. To minimize this potential bias, we focused on only breast cancer recurrence, not a disease-free survival as the primary measure of oncologic outcomes.

In conclusion, younger patients with breast cancer exhibited poorer pathological features than their older counterparts, such as a higher prevalence of high-grade tumors. However, younger age was not clearly associated with worse RFS or DRFS, particularly in aggressive subtypes, such as HER2-positive or TNBC. Nevertheless, younger age may be associated with a higher likelihood of LRR in HR-positive/HER2-negative patients, highlighting the need for more aggressive local treatment and vigilant surveillance in this group.

## Supplementary Material



## Data Availability

The datasets generated and/or analyzed during the current study are available in the Janghee Lee repository, which can be obtained by writing to doctorlee85@ewha.ac.kr.
