# Supplementary material for: Pathological characteristics and prognosis of very young patients with early breast cancer: a comparative analysis with older patients
Source: Acta Oncol. 2026 Jun 29;65:45584. doi: 10.2340/1651-226X.2026.45584 (PMC13324888; doi:10.2340/1651-226X.2026.45584)
Supplement: Supplementary file 1 [file AO-65-45584-s1.pdf]

Supplementary material has been published as submitted. It has not been copyedited, or typeset by Acta Oncologica

eTable 1. Multivariate analysis of risk factors for RFS and DRFS

	RFS		DRFS	
	Hazard ratio (95% CI)	*P*	Hazard ratio (95% CI)	*P*
Age: ≥55 vs ≤40	1.11 (0.74-1.66)	0.613	1.03 (0.65-1.62)	0.897
Breast surgery: BCS vs Mastectomy	1.23 (0.83-1.82)	0.304	1.46 (0.95-2.26)	0.087
Tumor size (mm): ≤20 vs >20	2.83 (1.84-4.35)	<0.001	3.43 (2.07-5.67)	<0.001
LN metastasis: Negative vs Positive	1.90 (1.26-2.87)	0.002	2.14 (1.34-3.41)	0.001
Subtype: HR-positive/HER2-negative vs HER2-positive	1.33 (0.83-2.13)	0.229	1.25 (0.75-2.08)	0.391
HR-positive/HER2-negative vs TNBC	1.80 (1.09-2.96)	0.021	1.39 (0.78-2.46)	0.261
HG: I or II vs III	1.03 (0.67-1.57)	0.900	0.92 (0.57-1.48)	0.737
LVI: Negative vs Positive	1.51 (1.01-2.25)	0.044	1.42 (0.91-2.21)	0.123
Chemotherapy: Not performed vs Performed	1.12 (0.47-2.66)	0.797	1.41 (0.50-3.99)	0.521

RFS, recurrence-free survival; DRFS, distant recurrence-free survival; CI, confidence intervals; BCS, breast-conserving surgery; LN, lymph node; HR, hormone receptor; HER2, human epidermal growth factor receptor 2; TNBC, triple negative breast cancer; HG, histologic grade; LVI, lympho-vascular invasion

eTable 2. Subgroup analysis of the effect of young age on RFS and DRFS

	RFS		DRFS	
	Hazard ratio (95% CI)	*P*	Hazard ratio (95% CI)	*P*
Tumor size (mm)				
≤20	1.45 (0.74-2.85)	0.281	0.99 (0.40-2.44)	0.985
>20	1.04 (0.65-1.64)	0.882	0.95 (0.58-1.58)	0.856
LN metastasis				
Negative	0.81 (0.44-1.50)	0.503	0.44 (0.18-1.06)	0.067
Positive	1.47 (0.91-2.39)	0.120	1.40 (0.83-2.37)	0.209
Subtype				
HR-positive/HER2-negative	1.62 (0.93-2.80)	0.089	1.35 (0.73-2.47)	0.341
HER2-positive	0.80 (0.36-1.75)	0.573	0.70 (0.28-1.77)	0.455
TNBC	1.07 (0.45-2.53)	0.881	0.80 (0.26-2.45)	0.692
HG				
I/II	1.07 (0.62-1.83)	0.812	1.23 (0.70-2.16)	0.468
III	1.17 (0.66-2.08)	0.593	0.83 (0.41-1.70)	0.612
LVI				
Negative	1.67 (1.02-2.74)	0.042	1.51 (0.85-2.69)	0.158
Positive	0.80 (0.43-1.49)	0.484	0.63 (0.30-1.33)	0.227

RFS, recurrence-free survival; DRFS, distant recurrence-free survival; CI, confidence intervals; LN, lymph node; HR, hormone receptor; HER2, human epidermal growth factor receptor 2; TNBC, triple negative breast cancer; HG, histologic grade; LVI, lympho-vascular invasion
